# Predictors and Outcomes of Salvaging the Corticospinal Tract After Thrombectomy in Basilar Artery Occlusion Stroke

**DOI:** 10.3389/fneur.2022.878638

**Published:** 2022-05-10

**Authors:** Dong-Seok Gwak, WooChan Choi, Yong-Won Kim, Dong-Hun Kang, Wonsoo Son, Yang-Ha Hwang

**Affiliations:** ^1^Department of Neurology, Dongguk University Ilsan Hospital, Goyang, South Korea; ^2^Department of Neurology, Kyungpook National University Hospital, Daegu, South Korea; ^3^Department of Neurology, School of Medicine, Kyungpook National University, Daegu, South Korea; ^4^Department of Radiology, School of Medicine, Kyungpook National University, Daegu, South Korea; ^5^Department of Neurosurgery, School of Medicine, Kyungpook National University, Daegu, South Korea

**Keywords:** pyramidal tracts, thrombectomy, basilar artery, stroke, ischemic stroke

## Abstract

**Background:**

Regional eloquence of brainstem structures may contribute to neurological status in basilar artery occlusion (BAO) stroke. The corticospinal tract (CST) which is vulnerable to BAO is important for motor activity. This study investigated the impact of CST salvage on outcomes and its associated factors in patients with BAO treated with thrombectomy.

**Methods:**

We retrospectively investigated 88 patients with BAO admitted ≤24 h after onset and presented with motor deficits and who underwent thrombectomy. Patients with a pre-stroke modified Rankin Scale (mRS) score of 4–5 who did not undergo baseline brain computed tomography angiography were excluded. CST salvage was evaluated using follow-up imaging (magnetic resonance imaging [MRI] or computed tomography when MRI was not available) after thrombectomy. A good outcome was defined as a 3-month mRS score of ≤2 or 3 if a patient's pre-stroke mRS score was 3. The associations between CST salvage and outcomes and clinical parameters were analyzed using logistic regression analyses.

**Results:**

Thirty-nine (44.3%) patients had CST salvage and the same number of patients had good outcomes. CST salvage was independently associated with a good outcome [adjusted odds ratio (aOR): 18.52, 95% confidence interval (CI): 4.31–79.67, *p* < 0.001]. After adjusting for confounders, atrial fibrillation (aOR: 3.92, 95% CI: 1.18–13.00, *p* = 0.026), location of occlusion (mid-BAO; aOR: 0.21, 95% CI: 0.06–0.72, *p* = 0.013), length of occlusion (involved segment of BAO <2; aOR: 4.77, 95% CI: 1.30–17.59, *p* = 0.019), and onset-to-puncture-time ≤180 min (aOR: 4.84, 95% CI: 1.13–20.75, *p* = 0.034) were significantly associated with CST salvage.

**Conclusion:**

CST salvage was associated with good functional outcomes in patients with BAO treated with thrombectomy. The presence of atrial fibrillation, location and length of BAO may predict CST salvage after thrombectomy, and rapid treatment with thrombectomy may protect this eloquent tract in these patients.

## Introduction

Ischemic stroke due to basilar artery occlusion (BAO) is a catastrophic condition that is associated with high rates of disability and mortality (1). Whether endovascular treatment (EVT) improves the prognosis of these patients remains unclear. Recent clinical trials on BAO treatment–BASICS (Basilar Artery International Cooperation Study) and BEST trial (Basilar Artery Occlusion Endovascular Intervention vs. Standard Medical Treatment)–failed to show superiority of EVT over standard medical treatment (2, 3). However, the high crossover rate in the BEST trial and a large number of screened patients treated outside of the trials might have affected the neutral results. Given the better outcome after EVT in per-protocol-treated patients in the BEST trial and in patients with a higher disease severity in the BASICS trial, EVT may be somewhat beneficial for patients with BAO who are likely to have poor prognosis with standard medical treatment. A more detailed analysis of prognostic factors is needed to achieve good outcomes after EVT in patients with BAO stroke.

Infarct location may play a critical role in functional impairment (4–6), especially in posterior circulation stroke where eloquent neural pathways are densely packed together (7). The corticospinal tract (CST) is a major neural pathway that is associated with post-stroke motor outcomes (8). It travels through the cerebral crus and the anterior part of the pons that are supplied by perforators originating from the basilar trunk, which accordingly is vulnerable to BAO. Sparing the CST through reperfusion therapy was associated with improved outcomes in anterior circulation stroke, independent of overall salvage of ischemic tissues (9, 10). Despite the encouraging result, this association has not been well-investigated in posterior circulation stroke. This study aimed to evaluate the impact of salvaging the CST on functional outcomes and its related factors in patients with BAO treated with EVT.

## Materials and Methods

### Study Population and Clinical Data Collection

**Figure 1:**
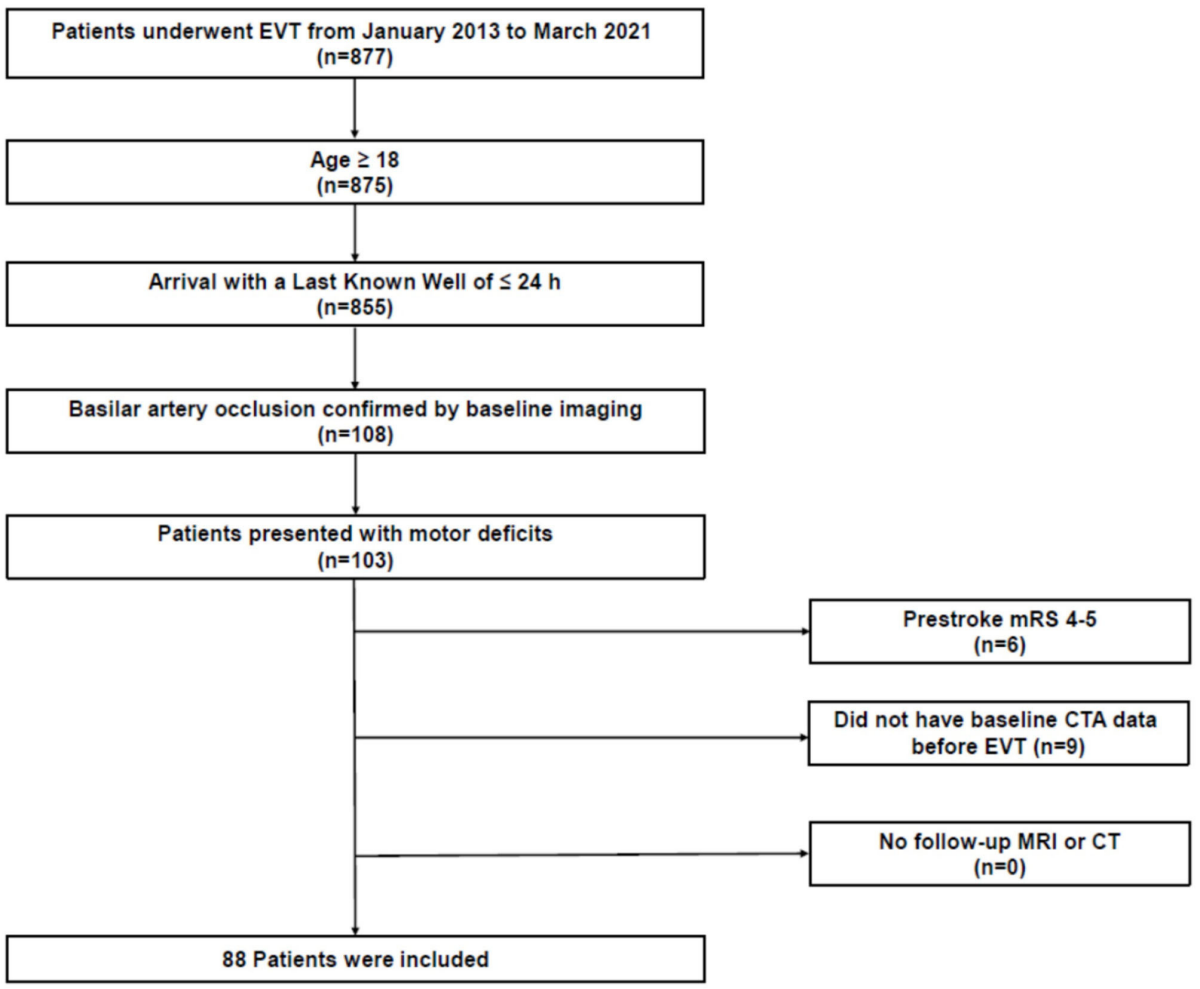
Study flowchart. CT, computed tomography; CTA, CT angiography; EVT, endovascular treatment; MRI, magnetic resonance imaging; mRS, modified Rankin Scale.

We retrospectively reviewed patients with acute ischemic stroke from a prospectively maintained institutional EVT registry database. Among 877 consecutive patients who were admitted to our center who underwent EVT between January 2013 and March 2021, patients who met the following criteria were included: (1) age ≥18 years, (2) admission within 24 h of the last known normal, (3) BAO confirmed by baseline imaging, and (4) baseline National Institutes of Health Stroke Scale (NIHSS) motor score (5a−6b) ≥1 (The clinical parameter was used for selecting patients whose CST is at risk of infarction). We excluded patients (1) with a pre-stroke modified Rankin Scale (mRS) score >3, (2) without available baseline computed tomography (CT) angiography (CTA) data before EVT, or (3) who had no follow-up magnetic resonance imaging (MRI) or CT (Figure 1). Baseline demographics and clinical characteristics were extracted from the institutional EVT registry or through a review of the electronic medical records. The study protocol was approved by the Institutional Review Board (approval number: KNUH 2021-09-014) and all procedures followed were in accordance with the institutional guidelines. The requirement for informed consent was waived due to the retrospective study design, data anonymity, and minimal risk to the study participants. Moreover, this study was performed according to the Strengthening the Reporting of Observational Studies in Epidemiology statement (11).

### Assessment of Images

**Figure 2:**
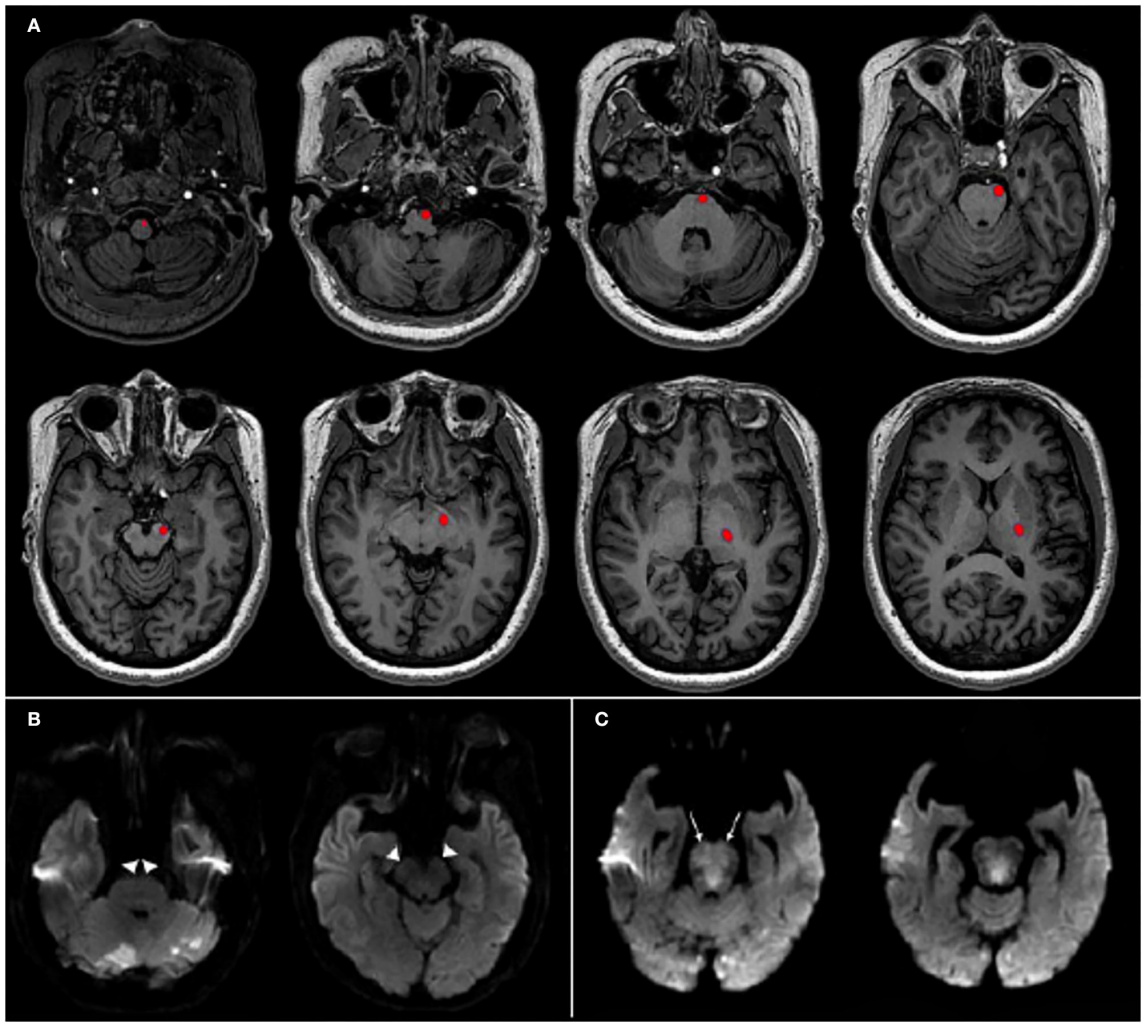
Templates of corticospinal tract (CST) and illustrative cases. **(A)** The pathways of CST from supratentorial to brainstem structures are shown in red at each level of the template maps. **(B)** A 69-y-old man who had a baseline National Institutes of Health Stroke Scale (NIHSS) score of 17 and distal basilar artery occlusion was treated by endovascular treatment (EVT). Time from symptom onset to groin puncture was 132 min and complete recanalization was obtained. Follow-up diffusion-weighted imaging after EVT revealed CST salvage (arrow heads). Final infarct volume was 24.3 mL and his 3-mo modified Rankin Scale (mRS) score was 2. **(C)** A 82-y-old woman who had a baseline NIHSS score of 27 and proximal to mid-basilar artery occlusion with onset-to-groin puncture time of 284 min was fully recanalized by EVT. Final infarct volume was 4.8 mL. However, follow-up diffusion-weighted imaging showed CST involvement on bilateral upper pons (arrows) and her 3-mo mRS score was 4.

The presence or absence of CST salvage was assessed on the follow-up diffusion-weighted imaging MRI (*n* = 72) after EVT or on CT (*n* = 16) when MRI was not available (median onset-to-follow-up image: 3.8 days, interquartile range [IQR], 1.2–5.3; The detailed imaging protocols are described in Supplementary Material). The templates of different layers showing CST including supratentorial and brainstem structures were derived from the atlas of MRI and CT (12, 13). Using the canonical templates, we identified the location of CST in each layer and then assessed whether the infarction involved the corresponding CST area by visual inspection (Figure 2) (14). Illustrative cases of patients with or without CST salvage are also presented in Figure 2. CST salvage was determined via independent evaluation by two experienced neurologists (D-SG and WCC) who were blinded to the clinical outcomes. Discrepancies were resolved by consensus (kappa index: 0.863 for all study participants, 0.889 for those with MRI, and 0.667 for those with CT). The baseline imaging scores based on CTA reflecting early ischemic change [posterior circulation Acute Stroke Prognosis Early CT Score [pc-ASPECTS] (15)], the extent of collateral flow [posterior circulation collateral score [PC-CS] (16)], and thrombus burden and location [posterior circulation CTA [pc-CTA] score (17) and Basilar Artery on CTA [BATMAN] score (18)] were measured in our cohort. The pc-ASPECTS is a 10-point scoring system where one point was subtracted for ischemic changes on each side of the cerebellum, occipital cortex, or thalamus, and two points were subtracted (per structure) for the midbrain and pons. PC-CS points were assigned as follows: one point for each patent cerebellar artery (posterior inferior cerebellar artery [PICA], anterior inferior cerebellar artery [AICA], and superior cerebellar artery [SCA]); one point for each patent posterior communicating artery (PCoA) if the diameter was smaller than the ipsilateral P1 segment of the posterior cerebral artery (PCA) and two points for each PCoA that had an equal or larger diameter than that of the ipsilateral P1 segment. The pc-CTA score allocated points in the absence of flow as follows: one point for either of the intracranial vertebral arteries, one point for each segment of the basilar artery (BA; the proximal segment was defined as the origin of the BA to the origin of the AICA, the middle segment was located between the AICA and SCA, and the distal segment was defined as the segment from the origin of the SCA to its rostral end); and one point for each PCA. The BATMAN score is a 10-point scoring system in which one point is assigned if either the intracranial vertebral artery was patent, each segment of the BA (proximal, middle, and distal segment) was patent, and each P1 segment of the PCA was patent, two points were assigned for the filling of each PCoA, and one point was allocated instead of two points if the PCoA was hypoplastic. The BA occlusion type was classified as truncal-type or branching-site occlusion. If the bifurcation site of the top of the BA was saved at the baseline CTA, it was classified as a truncal-type occlusion. Otherwise, it was classified as branching-site occlusion because of the association of the occlusion type with the etiology of acute large vessel occlusion (19, 20). Successful recanalization after EVT was defined as a modified treatment in cerebral ischemia grade 2b or 3 (21). Final infarct volumes and hemorrhagic transformation were assessed on follow-up MRI or CT scans. The final infarct volumes were measured using the imaging segmentation tool, ITK-SNAP software (http://www.tiksnap.org) (22). Hemorrhagic transformation was categorized as hemorrhagic infarction type 1, hemorrhagic infarction type 2, parenchymal hematoma type 1, and parenchymal hematoma type 2. Symptomatic intracerebral hemorrhage (sICH) was defined as parenchymal hematoma type 2 temporally related to neurological worsening ≥4 on the NIHSS score from baseline (23).

### Statistical Analysis

Data are presented as number (%), mean ± standard deviation, or median (IQR). Comparisons of variables between the patients with CST salvage (CST -) and CST involvement (CST +) were performed using the Student's *t*-test or Mann–Whitney *U* test for continuous variables and the Chi-squared test or Fisher's exact test for categorical variables. The association between CST salvage and clinical outcomes was analyzed using a multivariable logistic regression model. The primary outcome was a 3-month good functional outcome, defined as an mRS score of 0–2 or 3 if the patients' pre-stroke mRS score was 3 (i.e., clinical recovery to the status prior to the index stroke). Secondary outcomes included 3-month good-to-moderate functional dependence (mRS score, 0–3), 3-month functional independence (mRS score, 0–2), mortality at 3 months, hemorrhagic transformation, sICH, NIHSS score at day 7 or discharge, early neurological improvement (defined as either an NIHSS score at day 7 or discharge ≤1 or a difference between the NIHSS score at day 7 or discharge and at baseline ≥8) (10, 24), and final infarct volume. Due to the small sample size, a pre-specified multivariable model was constructed in which clinically important variables (age, sex, baseline NIHSS total score, stroke mechanism, atrial fibrillation (AF), onset-to-puncture time, modified treatment in cerebral ischemia grade, and final infarct volume) were entered as covariates, and a backward stepwise method was used for variable selection. Clinical and imaging parameters related to CST salvage were also analyzed using univariable and multivariable logistic regression analyses. The baseline CTA imaging scores were analyzed as either continuous variables or categorical variables that were dichotomized based on the literature [pc-ASPECTS of 8–10 (15), PC-CS of 6–10 (16), pc-CTA score of 0–2 (17), and BATMAN score of 7–10 (18)]. Likewise, time variables were analyzed as either continuous or dichotomized variables with various exploratory cut-off values. When constructing multivariable models, variables with *P* <0.10 from the univariable analysis were included. In case of overlapping variables [for example, continuous and dichotomized variables of pc-ASPECTS and time variables (onset-to-door, onset-to-puncture, and onset-to-recanalization time)], the most significant variables were retained. Moreover, variables that were highly related (location and length of BAO) were incorporated into separate models. Based on selected variables, backward stepwise elimination was performed to identify independent associated factors for CST salvage. Moreover, a sensitivity analyses were conducted to evaluate the relationship of CST salvage with clinical outcomes and associated factors in subgroups in whom CST salvage was assessed with MRI, which is more sensitive for detecting infarcted tissue compared to CT scan. Two-sided *P* < 0.05 were considered statistically significant, and all statistical analyses were performed using SPSS (version 27.0; IBM Corp, Armonk, NY).

## Results

Eighty-eight patients with BAO who were treated with EVT were enrolled in this study [mean age: 71.7 ± 9.7 years; 49 (55.7%) were men]. In 39 (44.3%) patients, post-interventional imaging revealed CST salvage. The clinical characteristics were not significantly different between the groups with CST- and CST+, except for the baseline NIHSS total and motor scores, stroke mechanism, and AF. The baseline NIHSS total and motor scores were lower in patients with CST- than with CST+ (total: 16.3 ± 8.1 vs. 20.5 ± 6.9, *P* = 0.010; motor: 7.4 ± 4.8 vs.9.5 ± 4.6, *P* = 0.034, respectively). Those with CST- were more associated with cardioembolic stroke, whereas those with CST+ were more associated with atherothrombotic stroke. Moreover, AF was more frequent in those with CST- than in those with CST+ (69.2 vs. 36.7%, *P* = 0.002; Table 1).

**Table 1:** Baseline characteristics according to the corticospinal tract salvage.

	**All (*n* = 88)**	**CST- (*n* = 39)**	**CST+ (*n* = 49)**	***P*** **-value**
Age, years	71.7 ± 9.7	71.0 ± 9.6	72.2 ± 9.8	0.562
Men	49 (55.7)	20 (51.3)	29 (59.2)	0.459
Baseline NIHSS total score	18.6 ± 7.7	16.3 ± 8.1	20.5 ± 6.9	0.010
Baseline NIHSS motor score	8.6 ± 4.7	7.4 ± 4.8	9.5 ± 4.6	0.034
Pre-stroke mRS				0.883
0–1	71 (80.7)	32 (82.1)	39 (79.8)	
2	4 (4.5)	2 (5.1)	2 (4.1)	
3	13 (14.8)	5 (12.8)	8 (16.3)	
Stroke mechanism				0.006
LAA	28 (31.8)	6 (15.4)	22 (44.9)	
Cardioembolism	51 (58.0)	29 (74.4)	22 (44.9)	
Other determined	1 (1.1)	1 (2.6)	0 (0.0)	
Undetermined	8 (9.1)	3 (7.7)	5 (10.2)	
Hypertension	54 (61.4)	23 (59.0)	31 (63.3)	0.681
Diabetes mellitus	25 (28.4)	9 (23.1)	16 (32.7)	0.322
Hyperlipidemia	40 (45.5)	21 (53.8)	19 (38.8)	0.158
Atrial fibrillation	45 (51.1)	27 (69.2)	18 (36.7)	0.002
MI or angina	11 (12.5)	4 (10.3)	7 (14.3)	0.748
Prior stroke	20 (22.7)	8 (20.5)	12 (24.5)	0.658
Smoking	20 (22.7)	6 (15.4)	14 (28.6)	0.143
SBP, mmHg	151.5 ± 30.9	151.3 ± 27.6	151.7 ± 33.5	0.957
DBP, mmHg	86.5 ± 20.9	85.9 ± 15.5	87.1 ± 24.5	0.778
Initial glucose, mg/dL	150.0 ± 40.3	143.5 ± 34.8	155.2 ± 43.9	0.178
Intravenous tissue-type plasminogen activator	31 (35.2)	14 (35.9)	17 (34.7)	0.907
Imaging variables				
pc-ASPECTS	8.0 (7.0–10.0)	9.0 (8.0–10.0)	8.0 (6.0–9.0)	<0.001
pc-ASPECTS 8–10	58 (65.9)	30 (76.9)	28 (57.1)	0.052
PC-CS	6.0 (4.0–7.0)	6.0 (5.0–7.0)	5.0 (4.0–7.0)	0.137
PC-CS 6–10	46 (52.3)	23 (59.0)	23 (46.9)	0.261
pc-CTA score	2.0 (2.0–3.0)	2.0 (1.0–3.0)	3.0 (2.0–4.0)	0.011
pc-CTA score 0–2	46 (52.3)	25 (64.1)	21 (42.9)	0.047
BATMAN score	5.0 (4.0–7.0)	6.0 (5.0–7.0)	5.0 (3.0–7.0)	0.036
BATMAN score 7–10	31 (35.2)	18 (46.2)	13 (26.5)	0.056
Occlusion type				0.019
Branching-site occlusion	66 (75.0)	34 (87.2)	32 (65.3)	
Truncal-type occlusion	22 (25.0)	5 (12.8)	17 (34.7)	
Occlusion site				
Distal BA	70 (79.5)	35 (89.7)	35 (71.4)	0.034
Mid-BA	35 (39.8)	6 (15.4)	29 (59.2)	<0.001
Proximal BA	18 (20.5)	3 (7.7)	15 (30.6)	0.008
PCA	42 (47.7)	18 (46.2)	24 (49.0)	0.792
Pcom (absence of or hypoplastic)	48 (54.5)	22 (56.4)	26 (53.1)	0.754
Number of occlusions in the BA segment				<0.001
1	55 (62.5)	34 (87.2)	21 (42.9)	
≥2	33 (37.5)	5 (12.8)	28 (57.1)	
mTICI				0.007
0–2a	12 (13.6)	1 (2.6)	11 (22.4)	
2b−3	76 (86.4)	38 (97.4)	38 (77.6)	
Time variables				
Onset-to-door time, min	176.0 (72.5–399.3)	113.0 (41.0–370.0)	202.0 (123.5–559.0)	0.435
Onset-to-puncture time, min	265.0 (160.3–485.0)	186.0 (117.0–438.0)	284.0 (204.0–662.0)	0.318
Onset-to-puncture time ≤180 min	25 (28.4)	19 (48.7)	6 (12.2)	<0.001
Onset-to-puncture time ≤360 min	54 (61.4)	26 (66.7)	28 (57.1)	0.362
Onset-to-recanalization time, min	339.0 (224.0–601.5)	248.0 (146.0–476.0)	373.0 (259.5–708.0)	0.239
Onset-to-recanalization time ≤180 min	16 (18.2)	13 (33.3)	3 (6.1)	0.001
Onset-to-recanalization time ≤360 min	49 (55.7)	25 (64.1)	24 (49.0)	0.156

*Variables are presented as mean ± standard deviation, median (interquartile range), or absolute number (proportion). BA, basilar artery; BATMAN score, basilar artery on computed tomography angiography score; CST, corticospinal tract; DBP, diastolic blood pressure; LAA, large artery atherosclerosis; MI, myocardial infarction; mRS, modified Rankin Scale; mTICI, modified treatment in cerebral ischemia; NIHSS, National Institutes of Health Stroke Scale; PCA, posterior cerebral artery; pc-ASPECTS, posterior circulation Acute Stroke Prognosis Early CT Score; PC-CS, posterior circulation collateral score; pc-CTA score, posterior circulation computed tomography angiography score; Pcom, posterior communicating artery; SBP, systolic blood pressure*.

A comparison of imaging parameters, including baseline imaging scores, the occlusion type and site, is shown in Table 1. Compared with the groups with CST+, the groups with CST- had higher pc-ASPECTS [median (IQR), 9.0 (8.0–10.0) vs. 8.0 (6.0–9.0), *P* < 0.001], lower pc-CTA scores [median (IQR), 2.0 (1.0–3.0) vs. 3.0 (2.0–4.0), *P* = 0.011] and higher BATMAN scores [median (IQR), 6.0 (5.0–7.0) vs. 5.0 (3.0–7.0), *P* = 0.036]. Those with CST- showed higher rate of branching-site occlusion than with CST+ (87.2 vs. 65.3%, *P* = 0.019). Patients with CST- had higher rate of distal BAO (89.7 vs. 71.4%, *P* = 0.034), lower rates of mid-BA occlusion (15.4 vs. 59.2%, *P* < 0.001), proximal BA occlusion (7.7 vs. 30.6%, *P* = 0.008), and occlusion in the multiple segments of BA (12.8 vs. 57.1%, *P* < 0.001). Successful recanalization was achieved more frequently in patients with CST- than in those with CST+ (97.4 vs. 77.6%, *P* = 0.007). With respect to time variables, the rates of onset-to-puncture time and onset-to-recanalization time within 180 min were higher in the CST- group than in the CST+ group (48.7 vs. 12.2%, *P* < 0.001; 33.3 vs. 6.1%, *P* = 0.001, respectively; Table 1).

Thirty-nine (44.3%) patients achieved good functional outcomes at 3 months. Patients with CST- had a significantly higher rate of 3-month good functional outcomes than those with CST+ (79.5 vs. 16.3%, *P* < 0.001, Table 2). Moreover, good to moderate functional dependence and functional independence at 3 months were higher in the groups with CST salvage than in the CST involvement group (94.9 vs. 34.7%, *P* < 0.001 and 69.2 vs. 14.3%, *P* < 0.001, respectively). Those with CST- had lower NIHSS scores on the day of discharge or day 7 [median (IQR), 3.0 (1.0–5.0) vs. 11.0 (7.0–26.5), *P* < 0.001] and tended to have higher rates of early neurological improvement (79.5 vs. 42.9%, *P* = 0.001). Regarding safety outcomes including 3-month mortality, any hemorrhagic transformation, and sICH, patients with CST salvage had significantly lower rates of adverse outcomes than patients with CST involvement, except for sICH. Furthermore, those with CST- had a smaller final infarct volume than those with CST+ [median (IQR), 6.5 mL (0.6–14.3) vs. 17.3 (5.3–46.6), *P* = 0.001, Table 2]. The associations of CST salvage and 3-month good functional outcomes remained statistically significant after adjusting for pre-specified potential confounders {adjusted odds ratio (aOR), 18.52 [95% confidence interval (CI), 4.31–79.67], *P* < 0.001; Table 3}.

**Table 2:** Patient outcomes.

	**All (*n* = 88)**	**CST- (*n* = 39)**	**CST+ (*n* = 49)**	***P*** **-value**
3-mo good functional outcome^a^	39 (44.3)	31 (79.5)	8 (16.3)	<0.001
3-mo mRS 0–3	54 (61.4)	37 (94.9)	17 (34.7)	<0.001
3-mo mRS 0–2	34 (38.6)	27 (69.2)	7 (14.3)	<0.001
3-mo mortality	15 (17.0)	1 (2.6)	14 (28.6)	0.001
Hemorrhagic transformation				0.083
HI type 1	12 (13.6)	4 (10.3)	8 (16.3)	
HI type 2	9 (10.2)	2 (5.1)	7 (14.3)	
PH type 1	7 (8.0)	1 (2.6)	6 (12.2)	
PH type 2	4 (4.5)	1 (2.6)	3 (6.1)	
Any hemorrhagic transformation	32 (36.4)	8 (20.5)	24 (49.0)	0.006
sICH	3 (3.4)	0 (0.0)	3 (6.1)	0.251
NIHSS score on day 7 or discharge	6.5 (3.0–14.5)	3.0 (1.0–5.0)	11.0 (7.0–26.5)	<0.001
Early neurological improvement	52 (59.1)	31 (79.5)	21 (42.9)	0.001
Final infarct volume, mL	11.0 (2.4–37.5)	6.5 (0.6–14.3)	17.3 (5.3–46.6)	0.001

*Variables are presented as median (interquartile range) or absolute number (proportion). CST, corticospinal tract; HI, hemorrhagic infarction; mRS, modified Rankin Scale; NIHSS, National Institutes of Health Stroke Scale; PH, parenchymal hematoma; sICH, symptomatic intracerebral hemorrhage. ^a^3-mo good functional outcome was defined as an mRS score of 0–2 or 3 if the patients' pre-stroke mRS score was 3*.

**Table 3:** Associations between the corticospinal tract salvage and clinical outcomes.

**Outcome^**a**^**	**Univariable analysis**	***P*** **-value**	**Multivariable analysis^**b**^**	***P*** **-value**
3-mo good functional outcome^c^	19.86 (6.71–58.79)	<0.001	18.52 (4.31–79.67)	<0.001
3-mo mRS 0–3	34.82 (7.47–162.38)	<0.001	24.70 (4.57–133.40)	<0.001
3-mo mRS 0–2	13.50 (4.72–38.58)	<0.001	11.98 (3.74–38.41)	<0.001
3-mo mortality	0.07 (0.01–0.53)	0.010	0.12 (0.01–1.04)	0.054
Any hemorrhagic transformation	0.27 (0.10–0.70)	0.007	0.37 (0.14–1.02)	0.054
sICH	NA	NA	NA	NA
Early neurological improvement	5.17 (1.98–13.51)	0.001	8.84 (2.27–34.46)	0.002

*mRS, modified Rankin Scale; NA, not applicable; sICH, symptomatic intracerebral hemorrhage. ^a^Results are reported as odds ratio (95% confidence interval) for the groups with corticospinal tract salvage compared to the groups with corticospinal tract involvement after endovascular treatment. ^b^Adjusted for age, sex, baseline National Institutes of Health Stroke Scale total score, stroke mechanism, atrial fibrillation, onset-to-puncture time, modified treatment in cerebral ischemia, and final infarct volume. ^c^3-mo good functional outcome was defined as an mRS score of 0–2 or 3 if the patients' pre-stroke mRS score was 3*.

Various clinical and imaging parameters were analyzed to identify factors associated with CST salvage. Before performing multivariable logistic regression analysis, potential factors were selected through the binary logistic regression analyses (Table 4). Baseline NIHSS total score over motor score and pc-ASPECTS as a continuous rather than dichotomized variable were selected. For thrombus burden measurement, pc-CTA score as a continuous variable over BATMAN score was selected. Moreover, for time variable, onset-to-puncture time within 180 min was selected. The location and length of BAO were entered in multivariable models 1 and 2, respectively. In the subsequent multivariable analyses, AF [aOR 3.92 (95% CI, 1.18–13.00), *P* = 0.026 in model 1], location of occlusion [mid-BAO; aOR 0.21 (95% CI, 0.06–0.72), *P* = 0.013], length of occlusion [involved segment of BAO <2; aOR 4.77 (95% CI, 1.30–17.59) *P* = 0.019], and onset-to-puncture time ≤180 min [aOR 4.84 (95% CI, 1.13–20.75), *P* = 0.034 in model 1] showed independent associations with the CST salvage after EVT (Table 4).

**Table 4:** Variables associated with corticospinal tract salvage after endovascular treatment.

**Variables**	**Univariable analysis: crude OR (95% CI)**	***P*** **-value**	**Multivariable analysis, model 1^**a**^: adjusted OR (95% CI)**	***P*** **-value**	**Multivariable analysis, model 2^**b**^: adjusted OR (95% CI)**	***P*** **-value**
Baseline NIHSS total score, per 1-point decrease	1.08 (1.02–1.14)	0.013	1.08 (0.99–1.18)	0.072	1.08 (0.99–1.17)	0.088
Baseline NIHSS motor score, per 1-point decrease	1.11 (1.01–1.21)	0.037	NA	NA	NA	NA
Stroke mechanism^c^	0.22 (0.08–0.63)	0.005	NA	NA	NA	NA
Branching-site occlusion	3.61 (1.19–10.94)	0.023	NA	NA	NA	NA
Atrial fibrillation	3.88 (1.59–9.48)	0.003	3.92 (1.18–13.00)	0.026	3.61 (1.09–11.95)	0.035
pc-ASPECTS	1.79 (1.29–2.49)	<0.001	1.43 (0.94–2.15)	0.092	1.45 (0.96–2.22)	0.079
pc-ASPECTS 8–10	2.50 (0.98–6.37)	0.055	NA	NA	NA	NA
pc-CTA score	0.61 (0.41–0.91)	0.015	NA	NA	NA	NA
pc-CTA score 0–2	2.38 (1.003–5.66)	0.049	NA	NA	NA	NA
BATMAN score	1.26 (1.01–1.58)	0.039	NA	NA	NA	NA
BATMAN score 7–10	2.37 (0.97–5.80)	0.058	NA	NA	NA	NA
Distal BA occlusion	3.50 (1.05–11.69)	0.042	NA	NA	NA	NA
Mid-BA occlusion	0.13 (0.04–0.36)	<0.001	0.21 (0.06–0.72)	0.013	NA	NA
Proximal BA occlusion	0.19 (0.05–0.71)	0.014	NA	NA	NA	NA
BA occlusion segment <2	9.07 (3.03–27.13)	<0.001	NA	NA	4.77 (1.30–17.59)	0.019
mTICI, 2b−3	11.00 (1.35–89.46)	0.025	13.74 (0.70–268.91)	0.084	12.33 (0.68–222.86)	0.089
Onset-to-puncture time ≤180 min	6.81 (2.36–19.65)	<0.001	4.84 (1.13–20.75)	0.034	4.30 (1.05–17.65)	0.043
Onset-to-recanalization time ≤180 min	7.67 (2.00–29.41)	0.003	NA	NA	NA	NA

*BA, basilar artery; BATMAN score, basilar artery on computed tomography angiography score; CI, confidence interval; mTICI, modified treatment in cerebral ischemia; NA, not applicable; NIHSS, National Institutes of Health Stroke Scale; pc-ASPECTS, posterior circulation Acute Stroke Prognosis Early CT Score; pc-CTA score, posterior circulation computed tomography angiography score; OR, odds ratio. ^a^Adjusted for the baseline NIHSS total score, stroke mechanism, branching-site occlusion, atrial fibrillation, pc-ASPECTS, pc-CTA score, distal BA occlusion, mid-BA occlusion, proximal BA occlusion, mTICI, and onset-to-puncture time ≤180 min. ^b^Adjusted for the baseline NIHSS total score, stroke mechanism, branching-site occlusion, atrial fibrillation, pc-ASPECTS, pc-CTA score, BA occlusion segment <2, mTICI, and onset-to-puncture time ≤180 min. ^c^Large artery atherosclerosis compared to the other stroke mechanisms*.

Similar results were observed in the sensitivity analysis in patients whose CST lesions were assessed with MRI. The baseline characteristics of this subgroup were presented in Supplementary Table 1. Patients with CST- had better clinical outcomes in terms of 3-month good functional outcomes [aOR, 17.54 (95% CI, 4.48–68.71), *P* < 0.001] and other secondary outcomes than patients with CST+ (Supplementary Table 2). Location (mid-BAO) and length of occlusion (involved segment of BAO <2) were still independently associated with CST in this subgroup, whereas pc-ASPECTS and successful reperfusion (modified treatment in cerebral ischemia grade of 2b−3) were newly detected as independent predictors of CST salvage (Supplementary Table 3).

## Discussion

In the present study, the CST could be salvaged by EVT in 44.3% of the patients with acute BAO. Moreover, sparing the CST was associated with a clinical benefit, including good functional outcome and early neurological improvement. Although several scoring systems with pretreatment imaging that weighted higher scores in brainstem lesions compared to other lesion locations have been developed for outcome prediction in patients with acute BAO treated with EVT (7, 25), to the best of our knowledge, this is the first study to evaluate the direct relationships between CST salvage and predictors and clinical outcomes in these patients. Our findings suggested that a clinical parameter (AF) and clot characteristics (location and length of BAO at baseline CTA) may enable early prognostication of CST salvage after EVT without advanced imaging, and that rapid initiation of EVT may protect this eloquent tract.

The strong association between the CST salvage and good clinical outcomes in our study indicated that the eloquence of certain brain areas could have profound effects on outcome after EVT (10, 26) and it could be explained by the fact that the intact CST was a predictor of independent walking after stroke (27). We found that the extent of BAO was associated with CST salvage. Longer clots may obstruct more perforating branches of the BA and consequently have a greater chance of inducing extensive ischemic damage in the brainstem. Moreover, the most vulnerable segment of BA to CST involvement was the mid-BA, where penetrating pontine arteries that supply the CST are located. These characteristics of BAO were robust predictors for CST salvage confirmed by sensitivity analysis. AF is also associated with CST salvage. In contrast to those with intracranial atherosclerosis, patients with AF were less likely to have underlying severe stenosis in the target artery, which may require a longer thrombectomy procedure time and additional rescue treatments such as balloon angioplasty or stenting (28). Furthermore, early initiation of EVT was linked to a higher chance of CST salvage. Of 25 patients with an onset-to-puncture time of ≤180 min, 19 (76.0%) achieved CST salvage after EVT, which is in line with the results regarding anterior circulation large-vessel occlusion stroke (10). However, these data should be interpreted with caution, as large heterogeneity exists in the infarct growth rate among patients with BAO and slow progressors may benefit from EVT beyond this time window (29).

The strength of this study is the identification of the mechanism of good prognosis in patients with acute BAO treated with EVT. Predictors of CST salvage may maximize the therapeutic effect of EVT in this patient population, and in-hospital workflow management to facilitate the rapid initiation of EVT is warranted. However, our study has several limitations. First, we included patients with premorbid mRS scores ≤3; thus, the patients in our study cohort were older and had more AF than in other study cohorts (2, 3). The generalizability of our study results to different study cohorts should be validated. Second, measurement of CST salvage/involvement was performed by direct visual interpretation. Although it could be easily applied in clinical practice and showed excellent interobserver agreement, quantitative assessment of CST salvage could not be performed using this method. Third, CT scan was used for evaluating CST salvage/involvement in 18.2% of the study participants, which is less sensitive in detecting infarcted lesions compared to MRI. However, the proportions of CST involvement in groups measured by CT was no less than those in groups measured by MRI [75.0% (12/16) vs. 51.4% (37/72), *P* = 0.085, respectively]. Moreover, due to the small number of the patients whose CST lesions were assessed with CT, whether the associations between CST salvage and clinical outcomes and the predictors of CST salvage were consistent in this subgroup could not be reliably tested (Supplementary Tables 1, 4). Fifth, the effect of different lesion locations within the CST on clinical outcomes could not be analyzed. As the CST runs down to the lower brainstem, the compactness of the tract increases. Thus, lesions of similar size in the CST may lead to different outcomes depending on the lesion location (30). Sixth, bias may have been introduced from differences in initial stroke severity (NIHSS score, pc-ASPECTS, and thrombus burden) between the CST- and CST+ groups when investigating the relationship of CST salvage and outcome, although potential confounders were adjusted. Further robust studies are warranted to confirm our knowledge of the effects of CST salvage. Seventh, the study enrollment period of 9 years might affect the study results given the changes in practice with the advance of EVT technology. However, the major study outcomes among groups sorted by 3-year-interval admission years were not significantly different (Supplementary Table 5). Eighth, a small proportion (16.3%) of patients with CST+ showed a good functional outcome in this study, which could be achieved partly due to peri-infarct areas compensating for the CST damage and facilitating motor recovery (31). Therefore, prognostic evaluation based solely on CST involvement should be avoided. Finally, whether the therapeutic time window may vary according to the clot length and location could not be evaluated. Further studies are required to develop more elaborate prognostic models that integrate imaging and time variables to identify EVT candidates in patients with BAO.

In conclusion, the occlusion site and length of BA at baseline CTA may predict CST salvage after EVT, and the CST could be protected by the rapid initiation of EVT in patients with acute BAO. Given that CST salvage is strongly associated with good functional outcomes, it may be considered as one of the potential therapeutic goals in patients with acute BAO who have undergone EVT; further well-designed studies are warranted.

## Data Availability Statement

The raw data supporting the conclusions of this article will be made available by the authors, without undue reservation.

## Ethics Statement

The studies involving human participants were reviewed and approved by Kyungpook National University Hospital. Written informed consent for participation was not required for this study in accordance with the national legislation and the institutional requirements.

## Author Contributions

D-SG established the study protocol, analyzed and interpreted the data, and drafted the manuscript. WCC, Y-WK, D-HK, and WS analyzed and interpreted the data and revised the manuscript for intellectual content. Y-HH established the study idea, analyzed and interpreted the data, and made revisions to the manuscript with intellectual input. All authors contributed to the article and approved the submitted version.

## Conflict of Interest

The authors declare that the research was conducted in the absence of any commercial or financial relationships that could be construed as a potential conflict of interest.

## Publisher's Note

All claims expressed in this article are solely those of the authors and do not necessarily represent those of their affiliated organizations, or those of the publisher, the editors and the reviewers. Any product that may be evaluated in this article, or claim that may be made by its manufacturer, is not guaranteed or endorsed by the publisher.
