# Supplementary material for: Predictors and Outcomes of Salvaging the Corticospinal Tract After Thrombectomy in Basilar Artery Occlusion Stroke
Source: Front Neurol. 2022 May 10;13:878638. doi: 10.3389/fneur.2022.878638 (PMC9127293; doi:10.3389/fneur.2022.878638)
Supplement: Supplementary file 1 [file Data_Sheet_1.docx]

Supplementary Material

**1 The imaging protocols evaluating the presence or absence of corticospinal tract salvage.**

MRI: Diffusion-weighted imaging (DWI) examinations were performed with scanners, SIGNA Architect, Discovery 750w, and SIGNA HDxt (GE Medical Systems, Milwaukee, WI). DWI parameters were as follow: repetition time of 6000 to 10 000 ms; echo time of 75 to 80 ms; field of view of 260 mm; image matrix number of 192×192, 192×160, or 160×160 pixels; b values of 0 and 1000 s/mm^2^; section thickness of 4.0 to 6.5 mm; and interslice gap of 0.4–1.5 mm.

CT: Computed tomography (CT) examinations were performed with scanners, Revolution EVO and Optima CT660 (GE Medical Systems, Milwaukee, WI). Brain CT parameters were as follow: field of view of 210mm, kV=120, various mAs as low as possible, helical scan with pitch of 0.5 to 1.0 as compliance of patient, 0.5mm of acquisition section thickness without overlap and reconstruction into 5mm thickness as default value.

# 2 Supplementary Table

**2.1 Supplementary Table 1. Baseline characteristics in patients whose CST lesions were assessed with MRI or CT.**

|  | | MRI (n=72) | | |  | CT (n=16) | | | |
| --- | --- | --- | --- | --- | --- | --- | --- | --- | --- |
|  | | CST- (n=35) | CST+ (n=37) | *P*-value | | CST- (n=4) | | CST+ (n=12) | *P*-value |
| Age, years | | 70.7 ± 8.9 | 71.0 ± 9.7 | 0.896 | | 73.5 ± 16.1 | | 75.9 ± 9.6 | 0.953 |
| Men | | 19 (54.3) | 22 (59.5) | 0.658 | | 1 (25.0) | | 7 (58.3) | 0.569 |
| Baseline NIHSS total score | | 16.6 ± 8.3 | 19.7 ± 7.2 | 0.098 | | 13.5 ± 5.1 | | 22.9 ± 5.3 | 0.013 |
| Baseline NIHSS motor score | | 7.6 ± 4.9 | 9.1 ± 4.7 | 0.196 | | 5.8 ± 2.4 | | 11.0 ± 4.0 | 0.030 |
| Pre-stroke mRS | |  |  | 0.973 | |  | |  | 1.000 |
| 0–1 | | 30 (85.7) | 31 (83.8) |  | | 2 (50.0) | | 8 (66.7) |  |
| 2 | | 2 (5.7) | 2 (5.4) |  | | 0 (0.0) | | 0 (0.0) |  |
| 3 | | 3 (8.6) | 4 (10.8) |  | | 2 (50.0) | | 4 (33.3) |  |
| Stroke mechanism | |  |  | 0.059 | |  | |  | 0.165 |
| LAA | | 6 (17.1) | 15 (40.5) |  | | 0 (0.0) | | 7 (58.3) |  |
| Cardioembolism | | 26 (74.3) | 18 (48.6) |  | | 3 (75.0) | | 4 (33.3) |  |
| Other determined | | 1 (2.9) | 0 (0.0) |  | | 0 (0.0) | | 0 (0.0) |  |
| Undetermined | | 2 (5.7) | 4 (10.8) |  | | 1 (25.0) | | 1 (8.3) |  |
| Hypertension | | 23(65.7) | 22 (59.5) | 0.584 | | 0 (0.0) | | 9 (75.0) | 0.019 |
| Diabetes mellitus | | 7 (20.0) | 14 (37.8) | 0.096 | | 2 (50.0) | | 2 (16.7) | 0.245 |
| Hyperlipidemia | | 20 (57.1) | 14 (37.8) | 0.101 | | 1 (25.0) | | 5 (41.7) | 1.000 |
| Atrial fibrillation | | 23(65.7) | 14 (37.8) | 0.018 | | 4 (100.0) | | 4 (33.3) | 0.077 |
| MI or angina | | 3 (8.6) | 6 (16.2) | 0.480 | | 1 (25.0) | | 1 (8.3) | 0.450 |
| Prior stroke | | 7 (20.0) | 10 (27.0) | 0.483 | | 1 (25.0) | | 2 (16.7) | 1.000 |
| Smoking | | 4 (11.4) | 12 (32.4) | 0.032 | | 2 (50.0) | | 2 (16.7) | 0.245 |
| SBP, mmHg | | 155.1 ± 26.4 | 152.2 ± 29.6 | 0.669 | | 118.8 ± 14.9 | | 150.1 ± 44.9 | 0.262 |
| DBP, mmHg | | 86.4 ± 15.4 | 87.2 ± 23.0 | 0.852 | | 81.3 ± 17.4 | | 86.5 ± 29.7 | 0.953 |
| Initial glucose, mg/dL | | 143.6 ± 31.2 | 160.6 ± 46.5 | 0.071 | | 144.8 ± 65.5 | | 138.7 ± 30.2 | 1.000 |
| IV tPA | | 14 (40.0) | 11 (29.7) | 0.360 | | 0 (0.0) | | 6 (50.0) | 0.234 |
| Imaging variables | |  |  |  | |  | |  |  |
| pc-ASPECTS | | 9.0 (7.0-10.0) | 8.0 (6.5-8.5) | <0.001 | | 9.5 (8.3–10.0) | | 8.0 (6.0–9.8) | 0.212 |
| pc-ASPECTS 8-10 | | 26 (74.3) | 21 (56.8) | 0.118 | | 4 (100.0) | | 7 (58.3) | 0.245 |
| PC-CS | | 6.0 (5.0-7.0) | 5.0 (4.0-7.0) | 0.208 | | 8.0 (7.0-9.8) | | 5.5 (4.3-8.0) | 0.103 |
| PC-CS 6-10 | | 19 (54.3) | 17 (45.9) | 0.479 | | 4 (100.0) | | 6 (50.0) | 0.234 |
| pc-CTA score | | 2.0 (2.0-3.0) | 3.0 (2.0-4.0) | 0.131 | | 1.0 (1.0-1.0) | | 3.0 (2.0-3.8) | 0.001 |
| pc-CTA score 0-2 | | 21 (60.0) | 17 (45.9) | 0.233 | | 4 (100.0) | | 4 (33.3) | 0.077 |
| BATMAN score | | 5.0 (5.0-7.0) | 5.0 (3.0-6.5) | 0.079 | | 8.0 (7.3-8.8) | | 6.0 (4.3-7.0) | 0.020 |
| BATMAN score 7-10 | | 14(40.0) | 9 (24.3) | 0.154 | | 4 (100.0) | | 4 (33.3) | 0.077 |
| Occlusion type | |  |  | 0.095 | |  | |  | 0.569 |
| Branching-site  occlusion | | 31 (88.6) | 27 (73.0) |  | | 3 (75.0) | | 5 (41.7) |  |
| Truncal-type  occlusion | | 4 (11.4) | 10 (27.0) |  | | 1 (25.0) | | 7 (58.3) |  |
| Occlusion site | |  |  |  | |  | |  |  |
| Distal BA | | 32 (91.4) | 28 (75.7) | 0.073 | | 3 (75.0) | | 7 (58.3) | 1.000 |
| Mid-BA | | 5 (14.3) | 20 (54.1) | <0.001 | | 1 (25.0) | | 9 (75.0) | 0.118 |
| Proximal BA | | 3 (8.6) | 10 (27.0) | 0.042 | | 0 (0.0) | | 5 (41.7) | 0.245 |
| PCA | | 18 (51.4) | 17 (45.9) | 0.642 | | 0 (0.0) | | 7 (58.3) | 0.088 |
| Pcom (Absence of or hypoplastic) | | 21 (60.0) | 22 (59.5) | 0.963 | | 1 (25.0) | | 4 (33.3) | 1.000 |
| Number of occlusions in the BA segment |  | |  | <0.001 | | |  | | 0.077 |
| 1 | | 30 (85.7) | 17 (45.9) |  | | 4 (100.0) | | 4 (33.3) |  |
| ≥2 | | 5 (14.3) | 20 (54.1) |  | | 0 (0.0) | | 8 (66.7) |  |
| mTICI | |  |  | 0.028 | |  | |  | 0.529 |
| 0-2a | | 1 (2.9) | 8 (21.6) |  | | 0 (0.0) | | 3 (25.0) |  |
| 2b-3 | | 34 (97.1) | 29 (78.4) |  | | 4 (100.0) | | 9 (75.0) |  |
| Time variables | |  |  |  | |  | |  |  |
| Onset-to-door time, min | | 101.0  (40.0-329.0) | 202.0  (123.5-559.0) | 0.455 | | 307.0  (91.8-646.0) | | 250.0  (107.3-579.8) | 0.953 |
| Onset-to-puncture time, min | | 170.0  (116.0-405.0) | 284.0  (198.0-662.0) | 0.332 | | 451.0  (199.5-704.0) | | 328.0  (213.0-665.0) | 0.862 |
| Onset-to-puncture time ≤ 180 min | | 18 (51.4) | 5 (13.5) | 0.001 | | 1 (25.0) | | 1 (8.3) | 0.450 |
| Onset-to-puncture time ≤ 360 min | | 25 (71.4) | 22 (59.5) | 0.286 | | 1 (25.0) | | 6 (50.0) | 0.585 |
| Onset-to-recanalization time, min | | 245.0  (144.0-465.0) | 342.0  (249.0-711.5) | 0.302 | | 541.0  (240.5-753.8) | | 413.5  (313.8-718.3) | 0.862 |
| Onset-to-recanalization time ≤ 180 min | | 12 (34.3) | 3 (8.1) | 0.006 | | 1 (25.0) | | 0 (0.0) | 0.250 |
| Onset-to-recanalization time ≤ 360 min | | 24 (68.6) | 21 (56.8) | 0.301 | | 1 (25.0) | | 3 (25.0) | 1.000 |

Variables are presented as mean ± standard deviation, median (interquartile range), or absolute number (proportion). BA, basilar artery; BATMAN score, basilar artery on computed tomography angiography score; CST, corticospinal tract; CT, computed tomography; DBP, diastolic blood pressure; IV tPA, intravenous tissue-type plasminogen activator; LAA, large artery atherosclerosis; MI, myocardial infarction; MRI, magnetic resonance imaging; mRS, modified Rankin Scale; mTICI, modified treatment in cerebral ischemia; NIHSS, National Institutes of Health Stroke Scale; PCA, posterior cerebral artery; pc-ASPECTS, posterior circulation Acute Stroke Prognosis Early CT Score; PC-CS, posterior circulation collateral score; pc-CTA score, posterior circulation computed tomography angiography score; Pcom, posterior communicating artery; SBP, systolic blood pressure.

**2.2 Supplementary Table 2. Associations between the corticospinal tract salvage and clinical outcomes (patients whose CST lesions were assessed with MRI, n=72)**

| Outcome^a^ | Univariable analysis | *P*-value | Multivariable analysis^b^ | *P*-value |
| --- | --- | --- | --- | --- |
| 3-mo good functional outcome^c^ | 14.50  (4.64-45.32) | < 0.001 | 17.54  (4.48-68.71) | < 0.001 |
| 3-mo mRS 0–3 | 40.00  (4.94-323.74) | 0.001 | 35.48  (4.14-303.68) | 0.001 |
| 3-mo mRS 0–2 | 10.71  (3.56-32.26) | < 0.001 | 11.72  (3.30-41.65) | < 0.001 |
| 3-mo mortality | NA | NA | NA | NA |
| Any hemorrhagic transformation | 0.25  (0.09-0.70) | 0.008 | 0.33  (0.11-0.99) | 0.048 |
| sICH | NA | NA | NA | NA |
| Early neurological improvement | 4.58  (1.54-13.62) | 0.006 | 9.26  (1.77-48.46) | 0.008 |

CST, corticospinal tract; MRI, magnetic resonance imaging; mRS, modified Rankin Scale; NA, not applicable; sICH, symptomatic intracerebral hemorrhage. ^a^Results are reported as odds ratio (95% confidence interval) for the groups with corticospinal tract salvage compared to the groups with corticospinal tract involvement after endovascular treatment. ^b^Adjusted for age, sex, baseline National Institutes of Health Stroke Scale total score, stroke mechanism, atrial fibrillation, onset-to-puncture time, modified treatment in cerebral ischemia, and final infarct volume. ^c^3-mo good functional outcome was defined as an mRS score of 0–2 or 3 if the patients’ pre-stroke mRS score was 3.

**2.3 Supplementary Table 3. Variables associated with corticospinal tract salvage after endovascular treatment (patients whose CST lesions were assessed with MRI, n=72)**

| Variables | Univariable analysis: crude OR (95% CI) | *P*-value | Multivariable analysis, model 1^a^: adjusted OR (95% CI) | *P*-value | Multivariable analysis, model 2^b^: adjusted OR (95% CI) | *P*-value |
| --- | --- | --- | --- | --- | --- | --- |
| Baseline NIHSS total score, per 1-point decrease | 1.05  (0.99-1.12) | 0.100 | NA | NA | NA | NA |
| Baseline NIHSS motor score, per 1-point decrease | 1.07  (0.97-1.18) | 0.194 | NA | NA | NA | NA |
| Stroke mechanism^c^ | 0.30  (0.10–0.91) | 0.033 | NA | NA | NA | NA |
| Branching-site  occlusion | 2.87  (0.81-10.21) | 0.103 | NA | NA | NA | NA |
| Atrial fibrillation | 3.15  (1.20–8.25) | 0.020 | NA | NA | NA | NA |
| pc-ASPECTS | 1.85  (1.28-2.68) | 0.001 | 1.56  (1.01-2.42) | 0.046 | 1.77  (1.18-2.65) | 0.006 |
| pc-ASPECTS 8-10 | 2.20  (0.81-5.98) | 0.122 | NA | NA | NA | NA |
| pc-CTA score | 0.72  (0.48-1.10) | 0.133 | NA | NA | NA | NA |
| pc-CTA score 0-2 | 1.77  (0.69-4.50) | 0.234 | NA | NA | NA | NA |
| BATMAN score | 1.24  (0.97-1.59) | 0.082 | NA | NA | NA | NA |
| BATMAN score 7-10 | 2.07  (0.76-5.70) | 0.157 | NA | NA | NA | NA |
| Distal BA occlusion | 3.43  (0.84-13.93) | 0.085 | NA | NA | NA | NA |
| Mid-BA occlusion | 0.14  (0.05-0.45) | 0.001 | 0.16  (0.04-0.58) | 0.006 | NA | NA |
| Proximal BA occlusion | 0.25  (0.06-1.01) | 0.052 | NA | NA | NA | NA |
| BA occlusion segment <2 | 7.06  (2.24-22.21) | 0.001 | NA | NA | 6.59  (1.86-23.35) | 0.003 |
| mTICI, 2b-3 | 9.38  (1.11-79.49) | 0.040 | 17.02  (1.22-237.76) | 0.035 | 11.61  (1.07-126.42) | 0.044 |
| Onset-to-puncture time ≤ 180 min | 6.78  (2.14-21.45) | 0.001 | 3.66  (0.89-15.10) | 0.073 | NA | NA |
| Onset-to-recanalization time ≤ 180 min | 5.91  (1.50-23.30) | 0.011 | NA | NA | NA | NA |
|  |  |  |  |  |  |  |

BA, basilar artery; BATMAN score, basilar artery on computed tomography angiography score; CI, confidence interval; CST, corticospinal tract; MRI, magnetic resonance imaging; mTICI, modified treatment in cerebral ischemia; NA, not applicable; NIHSS, National Institutes of Health Stroke Scale; pc-ASPECTS, posterior circulation Acute Stroke Prognosis Early CT Score; pc-CTA score, posterior circulation computed tomography angiography score; OR, odds ratio. ^a^Adjusted for the baseline NIHSS total score, stroke mechanism, branching-site occlusion, atrial fibrillation, pc-ASPECTS, pc-CTA score, distal BA occlusion, mid-BA occlusion, proximal BA occlusion, mTICI, and onset-to-puncture time ≤180 min. ^b^Adjusted for the baseline NIHSS total score, stroke mechanism, branching-site occlusion, atrial fibrillation, pc-ASPECTS, pc-CTA score, BA occlusion segment <2, mTICI, and onset-to-puncture time ≤180 min. ^c^Large artery atherosclerosis compared to the other stroke mechanisms.

**2.4 Supplementary Table 4. Outcomes of patients whose CST lesions were assessed with CT**

|  | All (n=16) | CST- (n=4) | CST+ (n=12) | *P*-value |
| --- | --- | --- | --- | --- |
| 3-mo good functional outcome^a^ | 3 (18.8) | 3 (75.0) | 0 (0.0) | 0.007 |
| 3-mo mRS 0–3 | 3 (18.8) | 3 (75.0) | 0 (0.0) | 0.007 |
| 3-mo mRS 0–2 | 2 (12.5) | 2 (50.0) | 0 (0.0) | 0.050 |
| 3-mo mortality | 8 (50.0) | 1 (25.0) | 7 (58.3) | 0.569 |
| Hemorrhagic transformation | |  |  | 1.000 |
| HI type 1 | 2 (12.5) | 0 (0.0) | 2 (16.7) |  |
| HI type 2 | 0 (0.0) | 0 (0.0) | 0 (0.0) |  |
| PH type 1 | 0 (0.0) | 0 (0.0) | 0 (0.0) |  |
| PH type 2 | 2 (12.5) | 0 (0.0) | 2 (16.7) |  |
| Any hemorrhagic transformation | 4 (25.0) | 0 (0.0) | 4 (33.3) | 0.516 |
| sICH | 2 (12.5) | 0 (0.0) | 2 (16.7) | 1.000 |
| NIHSS score on day 7 or discharge | 22.5  (7.8-42.0) | 5.5  (2.5-10.8) | 32.5  (21.0-42.0) | 0.008 |
| Early neurological improvement | 4 (25.0) | 2 (50.0) | 2 (16.7) | 0.245 |
| Final infarct volume, mL | 23.7  (1.1-80.2) | 0.5  (0.3-10.9) | 46.6  (4.5-108.1) | 0.020 |

Variables are presented as median (interquartile range) or absolute number (proportion). CST, corticospinal tract; CT, computed tomography; HI, hemorrhagic infarction; mRS, modified Rankin Scale; NIHSS, National Institutes of Health Stroke Scale; PH, parenchymal hematoma; sICH, symptomatic intracerebral hemorrhage. ^a^3-mo good functional outcome was defined as an mRS score of 0-2 or 3 if the patients’ pre-stroke mRS score was 3.

**2.5 Supplementary Table 5. Clinical outcomes sorted by admission years (3-year interval).**

|  | Total  (n=88) | Admission in 2013-2015  (n=26) | Admission in 2016-2018  (n=39) | Admission in 2019-2021  (n=23) | *P*-value |
| --- | --- | --- | --- | --- | --- |
| 3-mo good outcome ^a^ | 39 (44.3) | 14 (53.8) | 19 (48.7) | 6 (26.1) | 0.113 |
| mTICI 2b-3 | 76 (86.4) | 23 (88.5) | 36 (92.3) | 17 (73.9) | 0.123 |
| 3-mo mortality | 15 (17.0) | 2 (7.7) | 6 (15.4) | 7 (30.4) | 0.126 |

Variables are presented as absolute number (proportion). ^a^3-mo good functional outcome was defined as an mRS score of 0-2 or 3 if the patients’ pre-stroke mRS score was 3.
